# Transitions in the Careers of Competitive Swimmers: To Continue or Finish with Elite Sport?

**DOI:** 10.3390/ijerph17186482

**Published:** 2020-09-06

**Authors:** Malgorzata Siekanska, Jan Blecharz

**Affiliations:** Department of Psychology, Faculty of Physical Education and Sport, University of Physical Education in Krakow, al. Jana Pawla II 78, 31-571 Krakow, Poland; malgorzata.siekanska@awf.krakow.pl

**Keywords:** elite swimmers, career termination, career transition, athlete’s development, athletes’ health problems, supportive environment

## Abstract

An athletic career is a succession of stages and transitions (normative and non-normative), which may have decisive effects on either maintaining a satisfactory and/or successful course or deciding about premature career termination. The main purpose of this study was to identify differences between swimmers (M_age_ = 21.32, *SD* = 2.62) who after undergoing the transition from junior to senior level either: (a) continued their career hoping to improve their performance regardless of low success at the elite level—Group I; or (b) decided on premature athletic career termination—Group II. The criteria for inclusion included having undergone the transition from junior to senior level and having competed at elite level for at least a year (M = 4.14 years, *SD* = 1.74). The participants were administered a demographic survey and a structured interview. The results revealed differences in developmental stages. The exploration phase was longer (M_Group I_ = 5.8 yrs, *SD* = 2.04, M_Group II_ = 4.6 yrs, *SD* = 2.4; *Z* = −1.902, *p* = 0.057); the commitment phase was shorter (M_Group I_ = 3.6 yrs, *SD* = 1.3; M_Group II_ = 4.6 yrs, *SD* = 1.7; *Z* = −1.735, *p* = 0.083); the number of hours of structured practice were (M_Group I_ = 5975, *SD* = 2474; M_Group II_ = 7623, *SD* = 2660, Z = −1.928, *p* = 0.054); the number of perceived costs were (Group I = 22, Group II = 34; *Z* = −2.209, *p* = 0.0027); and the most often pointed benefits of a sporting career were (Group I—94% *health & physical fitness*; Group II—88% *personal growth & life skills*). Furthermore, essential inhibitors and facilitators of athletes’ development were identified. The findings of the study have practical applications for athletes, coaches, parents and sport psychology professionals. For instance, appropriate social support can help to prevent elite athletes’ premature career termination from professional sport during and after their normative transition from junior to senior level.

## 1. Introduction

The decision to continue or finish a career, when age does not pose any significant limitations but results are not satisfying anymore, is critical not only in the context of an athlete’s current situation but also the in overall perspective of an athlete’s life and values. Therefore, we were interested in the reasons why athletes decide to continue or finish their professional sport activity. It was assumed that autobiographical memory was not inaccurate since the research was conducted shortly after athletes’ decision concerning their future sport career.

One of the motives in conducting the research was that researchers favor studying successful athletes and marginalize less successful ones or those in crisis transitions ([1], p. 20).

An athlete’s sports career may seem to develop in a smooth and continuous way, but in fact it consists of specific phases, transitions and crises [2]. Researchers have identified a sequence of career developmental stages, not only in elite level athletes [3], but in talented performers in general [4,5]: initiation, development, mastery, and post-career phase. In the 1980′s, in contrast to the thanatological and gerontological approaches, transition models became popular (e.g, the Model of Human Adaptation to Transition) [6]. A transition was defined as “an event or non-event which results in a change in assumptions about oneself and the world and thus requires a corresponding change in one’s behavior and relationships” ([6], p. 5). Since transitions come with new circumstances, specific demands appear, related to practice, competitions, and to an athlete’s life in general. Changes usually take time, so athletic career transitions are perceived as a process of coping with a set of transition demands, not singular events [7,8]. A sports career is defined as a succession of stages and transitions, normative and non-normative, which may be decisive in either maintaining a satisfactory and/or successful course or prematurely discontinuing the career [9]. Normative transitions are predictable, anticipated, voluntary, and can be planned. Non-normative transitions are quite the opposite; they are unpredictable, unanticipated, involuntary, and cannot be planned [10]. Stambulova [9] identified the following six normative transitional periods of an elite athletic career: (1) the beginning of sport specialization, (2) the transition to more intensive training in the specific sports discipline, (3) the transition from junior to senior sport, (4) the transition from amateur to professional sports, (5) the transition from peak to the final stage, and (6) the transition to post-career. Contemporary sport transition scholars underline a holistic, lifespan developmental perspective on career transitions in sport contexts [11,12,13]. On the one hand an increased recognition of key transitional periods for performance excellence and psychosocial well-being in the life course of an athletic career has been recently observed; on the other hand, there are few studies still on within-career transitions in sport [14,15]. Scholars have broadened their focus beyond career termination, which seems aligned with recommendations on sport transitions [1,12]. Recently, there has been an increased focus also on contextual factors that are related to the athlete’s career development and transitions, i.e., environment and culture [16]. These factors contribute to the quality of the transitions [17] and influence the availability of career services advising on transitions out elite sport [16], as well as for dual-career development [18].

This study aims to contribute to the body of knowledge on career transitions in competitive swimming, which is not only one of the most popular but also one of the most time-consuming and demanding sports [19].

In their quest for accomplishments, elite swimmers progress through different developmental stages with the primary goal of improving performance, to be further confirmed by significant outcomes [20]. Usually, their career starts at the age of six, and it takes about ten years of deliberate practice to reach the elite level [21,22]. Before retirement, athletes usually stay at the top for a period of 5–15 years [23]. In swimming, men achieve peak performance later than women (24.2 ± 2.1 vs. 22.5 ± 2.4 years). However, the duration of the peak-performance window is similar for both sexes (2.6 ± 1.5 years) [24].

Beside outcomes (e.g., titles, medals, records), the duration of sport participation, from start to peak and finish, is one of the objective parameters that characterize a sports career. Subjective parameters include perceived benefits of sport participation (e.g., development through sport) and its costs (e.g., time, effort, health, money) [2]. The purpose of this study is to explore competitive swimmers’ perception of their career development and subjective career parameters and to identify differences between swimmers who—having undergone the transition from junior to senior level—either continued their career hoping to improve their performance, regardless of low success at the elite level, or resigned from competitive sport.

## 2. Materials and Methods

### 2.1. Ethical Issues

The research was carried out in accordance with the Helsinki Declaration. The authors did not know any of the participants. During an introductory meeting, participants were introduced to the idea of the study, and ethical issues were explained (voluntary participation, confidentiality in data treatment, and presentation). Additionally, written consent from each participant was obtained. The research project received approval from the Ministry of Science and Higher Education No. N RSA1 001951.

### 2.2. Procedure

The participants were recruited via sports institutions. Contacts were obtained from coaches. Swimmers who met all the criteria of inclusion were invited to take part in the study. The research was conducted by the first author in individual sessions (one-on-one), in places convenient for the participants, lasting approximately 60 min each. The study design used in the analysis included qualitative and quantitative measures [25]. The data were collected using a demographic survey and a structured interview. The researcher asked the same questions, in the same way, in the same order to all interviewees. Not only does this type of standardized interview facilitate analysis, but the responses obtained this way are easy to find and compare across individual interviews [26].

### 2.3. Participants

Thirty-four swimmers (M _age_ = 21.32, *SD* = 2.62), including 17 females (M _age_ = 21.53, *SD* = 2.9) and 17 males (M _age_ = 21.12, *SD* = 2.4), took part in the retrospective study.

All of the swimmers who participated in the research met the following criteria of inclusion: they had been identified as talents in the past; they had undergone a transition from junior to senior level; they had competed at elite level for at least one year (M = 4.14 years, *SD* = 1.74), i.e., participating in competitions at national and international level at least until 19 years of age.; their biggest accomplishments, i.e., personal best, winning at national (47% of participants) or international (53% of participants) competitions, had been achieved at junior level.

During the introductory meeting, two (out of 36) swimmers did not meet one of the inclusion criteria.

Most of the participants (79%) perceived their socio-economic status as very good (59%) or good (20%), and 17% described it as average. All participants were divided into two groups: (a) those who had continued their career hoping to improve their performance, regardless of low success at the elite level—Group I (7 females, 10 males); and (b) those who had decided on premature athletic career termination—Group II (10 females 7 males). Most of the participants (79%) had secondary education (Group I = 44%, Group II = 35%), 12% had Bachelor’s degree (6% in each group), and 9% had a Master’s degree (only Group II). As regards family environment, 32% of participants came from families with high sports achievements (no significant differences between groups).

### 2.4. Measures

The demographic survey consisted of the following items: age, sex, level of education, socio-economic status of family, sports achievements of family, highest accomplishment in swimming.

The interview was based on Gagné’s model—the Differentiated Model of Giftedness and Talent (DMGT) [5] in which developmental process (D) is influenced by two types of catalyst: Intrapersonal (I) and Environmental (E). These may exert—by their presence or absence—both positive (i.e., facilitating) and negative (i.e., inhibiting) influences. Since our focus was on gifted athletes’ development, and identification (in the past) as a talent in swimming was one of the crucial inclusion criteria, the interview questions related to: age of talent identification, main phases, pace of development, critical moments, persons perceived as important for career development, coaches perceived as important for career development; number of hours of structured practice; perceived benefits and costs related to participation in competitive swimming and sports career; essential facilitators and essential inhibitors of swimmers’ development; level of realization of one’s potential in essential areas of activity (in sport and out-of-sport); athletic career termination, i.e., premature resignation from competitive swimming (only in Group II). The interview consisted of both closed and open-ended questions. Examples of questions are as follows: *Your age when you began practicing swimming; Mark the number of hours per week (on average) you trained in swimming (at that level, i.e., exploration phase; commitment phase; proficiency phase); Mark the numbers of months per year (on average) you trained in swimming at that level, i.e., exploration phase; commitment phase; proficiency phase); Your age when you were identified as talented athlete; Your age when you began competitive swimming; What are/were your biggest sport accomplishments? Which of the factors contributed most to your development (facilitators of swimming development)? Mark on the scale (0–100%) the level of realization of your potential in essential areas of activity (in sport, out-of-sport). What were the reasons for your resignation from competitive swimming? (Group II only)*.

### 2.5. Analysis of Qualitative Data

The first step of analysis consisted of transcribing and then reading and re-reading the data in order to familiarize ourselves with it. In the next step, the raw data responses were content analyzed through a consensual procedure with the two researchers discussing and coming to consensus on the grouping of the raw data responses into meaningful subcategories and larger groupings. Like raw data responses were grouped first into subthemes, then subthemes were grouped into higher-order themes. To provide a full and clear report of the interview data analysis, in Tables 1, 3, 5, and 7 lower- and higher-order themes, as well as examples of direct quotes, are presented. In order to facilitate the reader’s understanding of the participants’ experience, exact quotations are provided [26].

Since the study is based on structured interview, many of the answers are easily quantifiable and thus subject to statistical analysis [26]. This facilitates analysis of data. Responses were easy to find and compare across individual interviews and across groups (cf. Tables 2, 4, 6 and 8), which is strictly related to the purpose of the research. On the other hand, the structured interview gives an opportunity for narrative comment.

The statistical analysis was conducted employing the Statistica 13.0 software (Statista, Hamburg, Germany). Basic descriptive statistical data were calculated for the analyzed quantitative variables, and the percentage values were calculated for the qualitative variables. Due to the small size of the sample, non-parametric statistics (i.e., the chi-square test) for the assessment of the relation between two nominal variables and the U Mann-Whitney test were employed. The results for which *p* was smaller than the accepted level of significance α = 0.05 were considered statistically significant.

The trustworthiness of the study was enhanced in four ways [27]. First, the authors did not cooperate with participants outside the research (i.e., before and after the data collection) and did not serve as sports psychology consultants (criterion of confirmability). Second, the pilot study was used to refine the interview protocol. Third, to be conservative, a threshold of 85% agreement was established and any uncertainty regarding categorization was discussed by the authors until resolved and an agreement had been made (criterion of dependability). Lastly, theoretical triangulation was ensured since multiple theories and perspectives were used to interpret the data (criterion of credibility). Moreover, a sports psychology consultant provided feedback as to whether the categories chosen by the researchers seemed to fit the data (criterion of transferability).

## 3. Results

Research showed no significant differences between groups regarding the sense of realization of one’s potential in sport (M_Gr.I_ = 76%, M_Gr.II_ = 79%, Chi^2^ = 1.167, *p* = 0.558), the sense of realization of one’s potential out-of-sport (M_Gr.I_ = 74%, M_Gr.II_ = 78%, Chi^2^ = 0.729, *p* = 0.543), and perceived benefits (Group I = 53, Group II = 46; *Z* = −1.428, *p* = 0.153) (Table 2).

Results showed some tendencies in developmental stages. In Group I, the exploration phase was longer (M_Group I_ = 5.8 yrs, *SD* = 2.04; M_Group II_ = 4.6 yrs, *SD* = 2.4; *Z* = −1.902, *p* = 0.057), and the commitment phase was shorter (M_Group I_ = 3.6 yrs, *SD* = 1.3; _MGroup II_ = 4.6 yrs, *SD* = 1.7; *Z* = −1.735 *p* = 0.083). This means that swimmers who continued their career (Group I) had more opportunities for sampling and deliberate play and that the phase of increased involvement and effort was shorter in comparison to those who quitted competitive swimming prematurely (Group II). Moreover, in Group II, swimmers went through crises more often (total number of crises: Group I = 16, Group II = 28) as they declared that they had thought of giving up sport earlier (Group I M _age_ = 17.6, Group II M _age_ = 16.6; *Z* = −3.078, *p* = 0.002).

With regard to the number of hours of structured practice, a tendency towards differences has been observed (Group I M = 5975 + −2474; Group II M = 7623 + −2660 Z = −1.928 *p* = 0.054).

On the basis of a qualitative analysis, five categories of benefits relating to participation in competitive swimming and a sports career were defined (Table 1). Most of the swimmers who continued their career (Group I) perceived health and physical fitness (94%), and opportunities to new experiences (88%) as the main benefits. In Group II personal growth & life skills (88%) and measurable rewards (59%) were mentioned most often (Table 2).

Based on a qualitative analysis, five categories of perceived costs were described (Table 3). Both groups pointed to lack of free time as the main cost (65%), moreover, in Group II injury & health problems, education, and mental consequences were named more often than in Group I (Table 4). The differences in the number of perceived costs of a swimmers’ sport career were statistically significant (Group I = 22, Group II = 34; *Z* = −2.209, *p* = 0.0027) (Table 4).

Furthermore, a number of essential facilitators (Table 5 and Table 6) and inhibitors (Table 7 and Table 8) of athletes’ development were identified.

A qualitative analysis revealed eight categories of swimmers’ sports career facilitators. Examples of the categories are presented in Table 5. In Group I the most commonly mentioned category was family support (65%) and this was significantly more frequently mentioned than in Group II (18%).

Another significant difference refers to natural abilities. This category appeared in 23% of participants in Group II while none of the participants in Group I mentioned it (Table 6). Moreover, an appropriate coach was perceived as the most important facilitator of swimmers’ development in both groups (20 participants in total (59%)).

As regards inhibitors, the results revealed a significant difference between the groups. Swimmers who continue their career (Group I) named more inhibitors than swimmers in Group II (Group I = 51, Group II = 44, *Z* = −2.652, *p* = 0.008) (Table 8). Based on the qualitative analysis, eight categories of inhibitors were identified. Table 7 includes all the categories with examples. In Group I, coach (65%) and family problems (65%) were mentioned most often. In Group II the category coach was pointed out by 76% of participants, and only 6% perceived family problems as an inhibitor of their sports development, the difference being statistically significant (*Z* = −3.251, *p* = 0.001).

In Table 9 reasons for athletic career termination are presented. Only one participant pointed out a single reason for career termination (accident that caused knee injury). The rest described their decision as a complex process influenced by many factors. It was not unsatisfactory accomplishments (29%) but the conflict between education and sport that was mentioned as the most common cause of quitting competitive swimming (41%).

No significant differences between group I and group II were found as regards: the number of significant others (family members *Z* = −0.041, *p* = 0.968; coaches *Z* = −1.103, *p* = 0.270) and financial situation (Chi^2^ = 1.167, *p* = 0.558).

## 4. Discussion

The study aimed to investigate competitive swimmers’ perception of their sports career development and to identify the differences between swimmers who, having undergone the transition from junior to senior level, either continued their career (Group I) or resigned from competitive sport (Group II).

As regards objective parameters, the participants in both groups presented a similar sporting level, since they competed at elite level and did not succeed. This means that they did not confirm their competences (i.e., medals, PR) at senior level and their biggest success (so far) was achieved at junior level. It was reasonable to focus on athletes’ subjective perception of the benefits and costs related to participation in competitive swimming and a sports career, since both groups represent crisis-transitions (low resources, excessive barriers and ineffective coping strategies) with a different secondary outcome [28]. Group II represents an unsuccessful transition associated with a premature dropout. As regards Group I, a ‘delayed’ successful transition with the help of effective support is possible.

The results showed that both groups declared similar, relatively high (74%–79%) sense of self-realization in sport and out-of-sport, which confirms that career satisfaction is determined by perceived benefits and a set of self-referenced criteria. This finding is in line with other research that confirmed that the level of achievement can be satisfying if it is related to perceived potential, and “athletes may be satisfied with nonelite careers if they value the developmental effects (e.g., benefits) of sport participation” ([2], p. 713). Research also confirmed that athletes found their sports career to be a meaningful life experience, even though they did not reach the elite level [8].

The study also revealed a significant difference between the groups as regards the perceived costs related to competitive swimming. Further analysis showed that these perceived costs might contribute significantly to premature athletic career termination. Health and academic problems are named both as costs of a sports career and as reasons for quitting competitive swimming (cf. Table 4 and Table 9). The qualitative analysis of the interviews revealed that swimmers perceived their athletic career to be in strong and reciprocal connection with their educational/academic career. Therefore, it seems important to focus on how to support student-athletes to develop coping strategies and to optimize the entire environment around them [13]. The cultural context should also be taken into account, since research shows that the sport’s system and dual career opportunities influence athletes’ career and development trajectories [18].

Other factors and circumstances relating to premature career termination, like unsatisfactory accomplishments or emotional and physical exhaustion, relate to burnout dimensions [29]. This shows that for swimmers who participated in this study sports career termination was not voluntary, as it was caused mainly by negative reasons (push factors) [30,31,32,33]. It is worth mentioning that elite level swimmers practice 20–30 h per week [19]. According to the perceptions of the athletes, it seemed that swimming left them with very little time and energy for other aspects of their lives. This might explain why swimmers reported a change in perspective (i.e., sport becoming less important than before, academic achievements becoming more important) [34].

As regards the process of swimmers’ sporting development, the present research revealed a tendency to differences in the number of hours of structured practice between the groups. Although several studies have emphasized the relationship between the number of training hours of an athlete and the performance level ultimately reached [35], it should be underlined that this refers not to any practice but only to appropriate quality training (deliberate practice, [36]). Those who resigned prematurely from competitive swimming had more hours of practice; however, the first phase of development, called the sampling years [22] or exploration stage [11], was significantly shorter and the second phase, which focused on developing skills and competences (i.e., the commitment stage), was significantly longer. This means that swimmers who resigned from competitive sport (Group II) invested more time and effort during their sports career and experienced more pressure. Together with low family support (Table 6), this could contribute to the fact that they went through crises more often than those who continued their career regardless of low success at the elite level.

Moreover, in both groups of participants, it was observed that social factors, such as negative relationship with the coach, or conflicts with parents and peers, play an important role as inhibitors of swimmers’ sport development (Table 7 and Table 8). Complementary to this is the observation that supportive environment and other social factors (e.g., good relationships and atmosphere) are perceived as the most important facilitators of athletes’ development (Table 5 and Table 6) [37,38]. This indicates that athletes who compete at the national level not only should have access to the best opportunities to improve, and to high-quality attention from coaches, but also must receive appropriate support from significant others in the out-of-sport environment. The family is one of the most important groups of reference in an athlete’s sport career. The current research shows that family can act as a facilitator (cf. Table 5 and Table 6) or inhibitor (cf. Table 7 and Table 8). On the one hand, a family can support the comprehensive development of an athlete, respect their autonomy, provide various forms of social support, form a motivational climate that aims at overcoming limitations but also at acquiring new skills [22,39]. On the other hand, a family can exert pressure on an athlete to achieve the best possible result at all costs by creating a motivational climate that aims at competition, comparison, and outperforming others [40,41].

The changes that take place in a sports career (normative, non-normative, quasi-normative) can stimulate athlete’s development, cause a crisis or be an adaptation to new forms (roles) of functioning, and a sports career itself can be perceived as a stage in life. It can be assumed that athletes who leave the sport in the absence of results have greater re-adaptive abilities and believe they can meet three basic psychological needs (autonomy, competence, and relatedness) outside sport [42].

Athletes who remain in the sport despite lack of results may fear re-adaptation (outside sport) or have positive illusions about their effectiveness and a belief that they will be able to improve their performance significantly [43].

Moments of transition can sometimes be dramatic and show that sports careers and life are not always linear but can experience dynamic and significant changes. The sense of identity of “being an athlete” can be so strong and emotionally marked that an athlete may fail to see the possibility of optimal functioning outside sport. Athletes may not be fully aware of their resources, including those acquired during a sports career.

It is, therefore, necessary to adopt a holistic approach to athletes and their careers (whole athlete approach) [44]. A sports career may be associated with a number of changes in areas such as the sport, education, family, and health. A whole athlete approach aims to equip athletes during their careers with skills and knowledge that will later facilitate their soft adaptation and transition from sport into a new environment, but also good mental health and openness to new experiences. Athletes should be aware of the problems they may encounter, the resources they have at their disposal, and how to make use of social support networks.

### 4.1. Strengths and Limitations

Trustworthiness of the study was achieved by direct quotes from the interviews. To facilitate transferability, the selection and characteristics of participants, data collection, and the process of analysis were described. There are also several limitations to this study, primarily related to the small size of the groups involved and the non-random selection of participants in both groups. Moreover, the study was retrospective in character, and the respondents’ answers may have been influenced by time. However, retrospective research designs are suggested to monitor the characteristics of young athletes aligned to future success [45].

It should also be emphasized that individual costs and benefits may have different impacts on the athlete’s development process, and some barriers may be easier to overcome than others. For example, a mental health problem can be more serious than a lack of free time.

### 4.2. Future research Directions

In further study, gender differences should be taken into account, as conflict between life goals (i.e., family vs. sports career) is one of the important determinants of premature career termination in female athletes [11].

## 5. Conclusions

The results obtained can contribute to a better understanding of the psychological and environmental factors that influence athletes’ careers, and that are advantageous to optimal talent development.

The findings from the study have practical applications for athletes, coaches, parents, psychologists, and sports professionals. A collaborative relationship between organizational and environmental factors (e.g., school—club communication) [46] and appropriate social support (especially family support) can help to prevent elite athletes’ premature career termination during and after their normative transition, within the transition from junior to senior level. As a result, it is important to educate parents, coaches, and teammates on the different roles they can play in providing the necessary social support to athletes undergoing normative within-career transitions [22,45,47]. Successful interventions should be based on assessment of athletes’ needs and on context-specific recommendations about what works and what does not work in helping athletes in different developmental stages (e.g., junior vs. senior) [48].

As regards the possible solutions for sub-elite athletes, five-step career planning [49] seems to be a good example of an intervention that is aimed at helping them to increase their self-awareness, to set realistic career goals bridging their past, present, and future, and to prepare in advance for the forthcoming transitions. Five-step career planning could be integrated into career assistance (CA) provided by career assistance programs (CAPs) or by private sports psychology consultants.

## Figures and Tables

**Table 1:** Categories of perceived benefits—Higher-Order and Lower-Order Themes from the Interview Data.

Category		Direct Quotes
Health & physical fitness (25)	Well-being (6) Physical development (8) Athletic silhouette (8) Body-awareness (3)	*Better health and good mood*; *Physical development and swimming skills*; *Neat silhouette and athletic body shape*; *Knowing your body and its abilities*;
Measurable benefits (13)	Financial benefits (7) Social recognition (6)	*Scholarships; Independent or partly independent subsistence; Financial gain*; *Fame & popularity; People recognize you*;
New experiences (22)	Meeting new people (12) Visiting new places (8) Unique experiences (2)	*Meeting new people in Poland and Europe; A lot of new friends*; *Traveling & sightseeing; Traveling and visiting interesting places*; *A wealth of experience and memories resulting from it*;
Personal growth & life skills (24)	Positive personal qualities (12) Useful skills (6) Self-realization (6)	*Self-discipline; Mental toughness*; *The habit of systematic work; Competitive sport gave me opportunity to learn how to cooperate*; *Self-realization; Comprehensive development*;
Satisfaction (15)	General satisfaction (8) Satisfaction from sport skills level (5) Satisfaction with achievements (2)	*Self-satisfaction; Feeling of satisfaction*; *Satisfaction out of the sport skills that your peers don’t have*; *Satisfaction from accomplishments*.

*Note.* Number of cases in parentheses.

**Table 2:** Perceived benefits of swimmers’ sport career—Comparison between groups.

Perceived Benefits	Group 1	Group 2
Health & physical fitness	16 (94%)	9 (53%)
Measurable benefits	3 (18%)	10 (59%)
New experiences	15 (88%)	7 (41%)
Personal growth & life skills	9 (53%)	15 (88%)
Satisfaction	10 (59%)	5 (29%)
TOTAL	53	46

**Table 3:** Categories of perceived costs—Higher-Order and Lower-Order Themes from the Interview Data.

Category		Direct Quotes
Lack of free time (22)	Limited time for social contacts (10) Limited time for other activities (7) Swimming as exceptionally time-consuming activity (5)	*Lack of time for friends; Lack of hanging out with friends*; *Lack of free time; Lack of time for other activities; Too little time to develop other interests and hobbies*; *Practicing swimming is very time-consuming; Workouts take all the time*.
Injury & health problems (13)	Sports injury (8) Health problems related to long-term competitive swimming (5)	*Injuries; Injuries that occur later; Recurring injuries*; *An adverse effect on health; Health deterioration; Limited contact with other disciplines deteriorates your general physical fitness*
Mental consequences (7)		*Feeling of underestimation; Mental problems—kids get depressed hearing their coach’s comments, having no support; You live as if “in a bubble”. It all revolves around the same people; Lack of carefree childhood;*
Education (8)		*It is problematic to cope with swimming and school; Sports development at the cost of school and other activities; Intellectual development and education is limited; You cannot learn and develop own interests; Schooling;*
Exhaustion (6)		*It takes a lot of effort; You feel constantly tired and don’t get enough sleep; Tiredness of traveling and being away from home; Swimming requires a lot of effort.*

**Table 4:** Perceived costs of swimmers’ sport career—Comparison between groups.

Perceived Costs	Group 1	Group 2
Lack of free time	11 (65%)	11 (65%)
Injury & health problems	5 (29%)	8 (47%)
Mental consequences	2 (12%)	5 (29%)
Education	2 (12%)	6 (35%)
Exhaustion	2 (12%)	4 (23%)
TOTAL	22	34

**Table 5:** Categories of facilitators—Higher-Order and Lower-Order Themes from the Interview Data.

Category		Direct Quotes
Appropriate coach (20)	Good approach to athletes (6) Motivational leader (5) Communication skills (5) Comprehensive competences (4)	*The right approach of the coach; Coach who was not authoritarian. In the beginning he was more like father who patiently explained everything. Then like a partner*; *Coach’s support and engagement that enhanced my motivation; You need to feel that the coach motivates you and encourages you. Then you want to train*; *Good communication with coach; Coach who just talked to us and explained*; *A great coach. He taught us how to be a good athlete. He knew how to speak to our hearts*. *He had amazing communication with young people. I would like to be like him*; *Appropriate/competent coaching staff*.
School & sport good cooperation (6)		*Good communication between teachers and a coach, mutual understanding*; *School that allows you to participate in swimming practice and competition*; *Teacher who appreciates sport achievements*; *Lack of problems with schooling;*
Intrinsic motivation (15)	Enjoying swimming (7) Setting & striving for goals (5) Determination (3)	*Your own interest in what you do, without coercion*; *Deriving pleasure from what you do, deriving pleasure from training, from competitions*; *Setting your most important goals and striving for them, staying focused on them without getting distracted*; *Desire to prove that you can do something, you can achieve something*; *Determination;*
Infrastructure and funds (17)	Infrastructure (9) Financial benefits (8)	*Infrastructure conditions*; *Access to a suitable pool; Appropriate conditions for training*; *Appropriate financial support*; *Competitions with financial rewards*; *Rewards and scholarships for good results;*
Feeling of success (12)	Sports development (5) Accomplishments (5) Better life (2)	*Significant progress—it gives you satisfaction and the feeling that it is worth to work hard*; *Getting better and better results*; *Good results and successful competing; Sport successes*; *I feel distinguished, being an athlete; I have more exciting life than my non-athlete peers*;
Family support (14)	Family atmosphere (8) Family involvement (6)	*When family accept what you are doing and believe that your involvement in swimming makes sense*; *Good atmosphere in the family*; *Feeling that you can always rely on your family*; *Family support during competitions*; *Motivation, support, and cheering from the family*; *When the whole family cheers; Parents’ financial support*;
Good relationships and atmosphere (9)	Relationships (5) Atmosphere (6)	*Friendship with people involved in similar activities; Good relationships in the group, in your team*; *Group atmosphere*; *Good atmosphere during practice;*
Natural abilities (4)		*Good health*; *Giftedness*; *Genes; Good health that protects you from injuries.*

**Table 6:** Essential facilitators of swimmers’ development—Comparison between groups.

Facilitators	Group 1	Group 2	Z	*p*
Appropriate coach	7 (41%)	13 (76%)	−1.587	0.113
School & sport good cooperation	3 (18%)	3 (18%)	0	1
Intrinsic motivation	5 (29%)	10 (59%)	−1.229	0.219
Infrastructure and funds	9 (53%)	8 (47%)	−0.138	0.890
Feeling of success	7 (41%)	5 (29%)	−0.691	0.489
Family support	11 (65%)	3 (18%)	−2.746	0.006
Good relationships and atmosphere	5 (29%)	4 (23%)	−0.383	0.702
Natural abilities	0 (0%)	4 (23%)	−2.098	0.036
Total	47	50	−1.073	0.283

**Table 7:** Categories of inhibitors—Higher-Order and Lower-Order Themes from the Interview Data.

Category		Direct Quotes
Coach (24)	Coach’s discouraging attitude (7) Coach’s low teaching competences (11) Conflicts with coach (6)	*Coach’s inappropriate attitude, lack of skills to motivate athletes*; *Coach’s discouraging approach, criticizing instead of saying what to improve, and how*; *The coach who doesn’t understand athlete’s needs and doesn’t assume a personalized approach*; *Bad training methods*; *Conflicts and lack of mutual understanding between a coach and an athlete; Lack of understanding and bad atmosphere.*
Schooling (11)		*It’s too big an effort to cope with school and competitive swimming*; *Problems with school duties*; *Bad grades at school*; *School learning*
Lack of motivation (10)		*Feeling bored with swimming*; *I lost my interest in swimming*; *Weak motivation and lack of goals*; *The monotony of swimming and hard workouts*
Lack of financial support (10)		*Lack of scholarship and funds*; *Lack of money*; *Limited access to financial support*; *Financial problems;*
Bad company (10)		*Spending too much time with people who don’t practice sport*; *Adverse influence of other people*; *Inappropriate company and temptations, like alcohol*; *Bad influence of friends and peers;*
Health problems (12)		*Poor physical condition*; *Illness*; *Injuries; Health and injuries*;
Family problems (12)	Bad family atmosphere (5) Lack of support and interest (7)	*Conflicts with parents*; *Family problems and lack of understanding*; *Family atmosphere*; *Parents were uninterested and unsupportive; Parents didn’t support. They were indifferent;*
Bad group atmosphere (6)		*Bad atmosphere in the group*; *Inappropriate atmosphere in the group and lack of someone to compete with*; *My friends quit swimming and it changed the atmosphere and decreased my motivation*; *Relations among swimmers.*

**Table 8:** Essential inhibitors of swimmers’ development—Comparison between groups.

Inhibitors	Group 1	Group 2	Z	*p*
Coach	11 (65%)	13 (76%)	−0.450	0.652
Schooling	7 (41%)	4 (23%)	−0.824	0.410
Lack of motivation	3 (18%)	7 (41%)	−1.483	0.138
Lack of financial support	7 41%)	3 (18%)	−0.931	0.352
Bad company	5 (29%)	5 (29%)	−0.293	0.769
Health problems	3 (18%)	9 (53%)	−1.599	0.110
Family problems	11 (65%)	1 (6%)	−3.251	0.001
Bad group atmosphere	4 (23%)	2 (12%)	−0.532	0.595
Total	51	44	−2.652	0.008

**Table 9:** Reasons for athletic career termination (Group II).

Reasons	Number and Percentage of Participants	Direct Quotes
Education	7 (41%)	*I was a student of a technical university and my teachers didn’t understand my situation as a competitive athlete*; *I wanted to focus on my school exams*; *I didn’t want to associate my further education with sport*; *I didn’t want to study at a sports university. I’ve decided to study economics and to focus on my education.*
Coach	5 (29%)	*My coach had too many obligations and he stopped getting involved*; *The coach exerted too much pressure*; *Bad relationship with the coach*; *Lack of mutual understanding with the coach.*
Unsatisfactory accomplishments	5 (29%)	*Lack of success*; *The results did not improve*; *It wasn’t like it used to be. Although I tried, I was not good at the competition anymore*; *Lack of results improvement*
Health problems	4 (23%)	*Injury*; *Health*; *Sinus problems caused by water and chlorine*; *Knee injury caused by skiing accident*
Emotional exhaustion	4 (23%)	*Emotional burnout*; *Disappointment and discouragement*; *I felt mentally burned out*; *Feeling discouraged*
Physical exhaustion	3 (18%)	*I was afraid of tremendous effort*; *Weariness and fatigue caused by swimming practice*; *Monotony and energy drain caused by swimming, and the number of workouts*
